# Cynical Hostility, Intimacy and Relationship Satisfaction: The Role of Depressive Symptoms

**DOI:** 10.3390/bs14121160

**Published:** 2024-12-03

**Authors:** Dikla Segel-Karpas, Roi Estlein, Roni Elran-Barak

**Affiliations:** 1 Department of Gerontology, University of Haifa, Haifa 3498838, Israel; 2 School of Social Work, University of Haifa, Haifa 3498838, Israel; restlein@univ.haifa.ac.il; 3 School of Public Health, University of Haifa, Haifa 3498838, Israel; relranbar@univ.haifa.ac.il

**Keywords:** cynical hostility, intimacy, relationship satisfaction, vulnerability–stress–adaptation

## Abstract

Background: An individual’s own and their perceived partner cynical hostility are conceptualized as vulnerability factors, decreasing couples’ intimacy and relationship satisfaction. The perceived partner cynical hostility may be especially harmful when distress is high. Method: Longitudinal data were collected in two waves (during and after the COVID-19 lockdown), relying on the respondents’ self-reports. Results: Intimacy mediated the association between perceived partner cynical hostility and relationship satisfaction in both waves. The association between perceived partner cynical hostility and intimacy was stronger when depressive symptoms were higher. No longitudinal effects were found. Conclusions: The perceived partner cynical hostility could be a meaningful vulnerability factor, hampering the ability to establish intimacy and, in turn, relationship satisfaction. The toll may be greater when individuals experience greater vulnerability.

## 1. Introduction

The COVID-19 pandemic not only added significant stress to the lives of individuals, couples, and families due to the meaningful health threat, but also altered their daily routines. As governments sought ways to respond to the pandemic, stay-at-home orders and lockdowns were enforced in many countries around the world, and couples and families found themselves spending more time together in the confined spaces of their homes. In this enforced situation, the intimate romantic partnership was crucial as either being a source of support and resilience or generating further stress with additive deleterious effects on couple and individual well-being.

To understand the varied outcomes this unusual situation may have had for different couples, we draw on the COVID-19-adpated Vulnerability–Stress–Adaptation model [1,2] which focuses on situational-, individual-, and couple-level variables. We identify the perceived own and romantic partner’s cynical hostility, that is, the belief that people are a source of wrongdoing [3], as a possible vulnerability factor that gained prominence during the joint lockdown. We examine the hypothesis that cynical hostility harmed the couple’s ability to establish intimacy within the stressful experience of the global pandemic, thus altering the adaptive processes, resulting in reduced relationship quality in the form of couple satisfaction and intimacy. We further argue that perceived partner cynical hostility would have taken a greater toll on the potential to gain intimacy when one’s emotional needs were greater. That is, one’s distress could act as an added vulnerability factor (that can be either enduring or situational, resulting from the pandemic), that amplifies the inability of couples with higher levels of cynical hostility to achieve intimacy in the relationship.

The data for this study were collected during the first lockdown enforced in Israel through March and April 2020, and again in early June 2020 after most of the restrictions were relaxed. The long lockdowns forced severe stay-at-home orders and caused meaningful distress in the general population [4].

### 1.1. Relationship Satisfaction and Intimacy

The importance of relationship satisfaction for individuals’ and couples’ health and well-being has been documented in numerous studies. As a marker of couple relationship quality, findings suggest that relationship satisfaction is associated with physical health [5], mental health [5,6], and even mortality [7]. Relationship satisfaction is associated with both couple- and individual-level factors. On the couple level, relationship satisfaction is associated with the nature (positive/negative) of the beliefs and expectations with which partners enter their marriage [8]; the interaction between positive and negative affect in the relationship, especially in times of conflict [9,10]; and the ways partners interact to routinely maintain their partnership by constructing their sense of togetherness [11]. In terms of individual factors, attachment orientation [12] and personality traits [13], particularly neuroticism [14,15], are predictive of relationship satisfaction. Further exploring the factors that contribute to relationship satisfaction is important because identifying such factors can help professionals to assist couples to increase relationship contentment and avoid behaviors that may have the opposite effect [16].

The Vulnerability–Stress–Adaptation (VSA) model [1] is a useful framework to examine factors that contribute to relationship satisfaction or distress, particularly in times of stress such as the COVID-19 global pandemic. According to the model, couple relationship satisfaction is affected by stressful events that the couple has to endure. Couples’ ability to cope with stress is affected both by their ‘enduring vulnerabilities’, which can include certain personality traits or tendencies, and the characteristics of the stressful event itself. Enduring vulnerabilities and the stressful event shape couples’ adaptive processes, which refer to the ways and means that couples employ in order to cope with the stress and its related conflict. In turn, the adaptive processes affect partners’ perceptions of their relationship quality, and as a result, its stability.

Applying the VSA model to the COVID-19 global pandemic, Pietromonaco and Overall [2] suggested that the COVID-19 pandemic was a context that generated multiple potential stressors, ranging from unemployment to homeschooling and overcrowded households. These stressors may interfere with couples’ adaptive processes, which can include the support they receive and provide, or the intimacy they manage to achieve. Thus, the COVID-19-related stressors may have negatively affected the quality of couples’ relationship and stability. Pre-existing and enduring vulnerabilities, such as depression, personality traits, or insecure attachment orientation can exacerbate the negative effects of the stressors and impede adaptive processes.

In this study, we focus on couple relationship satisfaction as an indicator of relationship quality [8,9,17], and conceptualize emotional intimacy as an adaptive process. We examine how one’s own and perceived partner cynical hostility serve as an enduring vulnerability factor that hampers the ability to gain intimacy.

### 1.2. Cynical Hostility and Couple Intimacy

The interpersonal theory of personality [18] provides a useful framework for examining how cynical hostility impacts social interactions. This theory views personality as a set of recurring interpersonal situations that define an individual’s social dynamics. People’s expectations and behaviors prompt reactions from others that often reinforce those initial expectations. In individuals with high levels of cynical hostility, negative expectations lead to behaviors that evoke adverse responses from social partners, creating a reinforcing “vicious cycle” of hostility [19,20].

Within a romantic partnership, intimacy refers to the feeling of connectedness and closeness between partners [21]. Intimacy is thought to be achieved by a reciprocal communication process, in which self-disclosure of one partner is met by responsiveness or empathy by the other [22,23]. Thus, the ability to achieve intimacy within a romantic relationship engages the two partners. This can be significantly hampered by one’s own and their partner’s insecurities, including cynical hostility.

Hostility is a social–cognitive schema, defined as an “attitude toward others, consisting of enmity, denigration, and ill will’’. Cynical hostility, a subset of hostility, is marked by a mistrust of others and the belief that people are driven by selfish motives [24]. Accordingly, it does not necessarily imply that others intend to cause harm, but rather that they are self-centered and indifferent to the well-being of those around them [20]. Hostility, along with its cynical aspect, belongs to a broader category that also encompasses anger and aggression. While anger represents an emotional response and aggression reflects a behavioral tendency, cynical hostility functions as a cognitive component, shaping how individuals perceive others and interpret their actions [20,24]. Cynical hostility is thought to impede one’s social relationships for several reasons. First, the basic mistrust in others may make it more difficult for individuals with high levels of cynical hostility to reach for social support and to establish and maintain intimacy [25]. Second, the expression of cynical hostility could make individuals high in cynical hostility less attractive counterparts [19,25,26] and hinder their partners’ attempts to seek support or intimacy from them. At the same time, their partner may be less willing to offer support or to positively respond to their support-seeking attempts. Finally, individuals with high levels of cynical hostility may be more prone to having interpersonal conflicts and experience disrespect from others. By expressing mistrust, they may offend others, making their social interaction unpleasant, and elicit negative behaviors from their social counterparts [19].

Indeed, empirical studies indicate that cynical hostility is associated with a poorer social life [20]. Using a series of cross-national, longitudinal, and experimental studies, Stavrova et al. [19] found a reciprocal relationship between cynical hostility and the experience of disrespect. Using longitudinal data and a cross-lagged design, Segel-Karpas and Ayalon [25] found that cynical hostility has a positive effect on loneliness, and at the same time, loneliness is a predictor of cynical hostility, suggesting that individuals who are high in cynical hostility struggle to secure the social connections they desire. In a longitudinal study of couples, the wife’s hostility was predictive of her husband’s loneliness, and the effect was partially mediated by the husband’s contact with his friends. The wife’s contact and support from her friends mediated the association between her own cynical hostility and loneliness [26]. Similarly, when examining cynical hostility and mental health in couples, the wife’s cynical hostility was associated with her husband’s mental health, but not vice versa [27]. Other studies also suggest that individuals with a higher level of cynical hostility both provide and receive less social support, experience greater strain, including from their offspring [28], or elicit calloused responses in times of need [29]. Individuals with high levels of cynical hostility also perceive their friends as less friendly and less supportive than individuals with low levels of cynical hostility, report strained relationships with their adult children [28], and experience greater anxiety when they are expected to provide support [30]. They are also more likely to interpret others’ facial expressions in a manner that corresponds with their negative expectations, taking disgust for anger, and happiness for neutrality [31]. Finally, and most relevant to intimacy, individuals with high levels of cynical hostility experience increased physiological reactivity in situations involving self-disclosure [32] as they may perceive these situations as threatening or uncomfortable. Their deep mistrust of others leads them to view sharing personal information as a risk, potentially exposing them to manipulation or betrayal. This mistrust makes them hyper-vigilant, increasing stress levels and eliciting physiological responses such as elevated heart rate and cortisol level [32].

Despite the importance of the committed romantic dyad as a most fundamental social connection, and despite the meaningful associations between cynical hostility and the ability to form and enjoy good social relationships, only relatively few studies have examined the relationship between cynical hostility and outcomes for couples. Using a longitudinal design following individuals for 11 years, higher levels of cynical hostility were predictive of divorce, separation, or not being married [33]. Following a sample of 95 couples over a period of 18 months, the husbands’ cynical hostility was found to be concurrently associated with their own, but not their wives’ marital adjustment, whereas the wives’ cynical hostility was associated with both their own and their partner’s marital adjustment. The results of the prospective analysis suggested that it was the wives’ anger (an affective component of hostility), rather than cynical beliefs (a cognitive component) that affected the husbands’ and wives’ marital adjustment [34]. However, in a similar design, following a sample of 53 newlyweds over a period of 3 years, the findings showed that the husbands’ cynical hostility was predictive of both their own and their wives’ deterioration in marital quality [35]. The authors suggest that the reduced marital quality reported by the husbands could be the result of a self-fulfilling prophecy, reflecting their negative expectations from social relationships. The husbands’ suspicious and conflictual behavior could be responsible for the reduced martial quality experienced by the wives [35]. Further support for the link between cynical hostility and relational distress in couples is provided in another study that found that high levels of hostility were related to lower relationship satisfaction and less agreement in relationship decisions. Surprisingly, it was unrelated to affective expression and the disclosure of emotional experiences [36].

In terms of the implications of cynical hostility on physiological health, Smith et al. [37] found that not only is one’s own cynical hostility related to inflammation, but so is one’s spouse’s cynical hostility. Cynical hostility in husbands was also found to result in their own and their spouse’s greater physiological reactivity during marital conflicts [38], and their own physiological reactivity under conditions of evaluative threat [39].

Taken together, studies suggest that cynical hostility takes a meaningful toll on one’s ability to engage in satisfying social relationships in general, and romantic partnerships in particular. We suggest that the negative outcomes for couples may be partially explained by the inability of partners with high levels of cynical hostility to establish intimacy, which is a significant adaptive process for couples. Their overall lack of trust in others could hinder their ability for self-disclosure that is necessary to achieve intimacy. Moreover, their hostile attitudes may make such partners less responsive counterparts [22], which also harms the ability to create intimacy within the intimate relationship. Thus, we hypothesize that (H1) intimacy will mediate the association between (H1a) one’s own and (H1b) their perceived partner cynical hostility and relationship satisfaction.

### 1.3. The Role of Depressive Symptoms

We further suggest that the deleterious effect of perceived partner cynical hostility can be accentuated by the experience of depressive symptoms. Given the complexity of the COVID-19 lockdown, and the emotional toll it took on individuals and families [40], we sought to examine whether emotional distress may exacerbated the negative impact of cynical hostility on relationship outcomes. According to the interpersonal perspective on depression, depressed individuals tend to communicate in ways that elicit rejection and deter others, and thus, in a reciprocal process, this results in greater depression (for a review, see [41]). The deleterious effects of depression on social relations are also apparent in the romantic dyad. Previous studies suggested a link between depressive symptoms and relationship distress ranging from dissatisfaction to conflict [42,43,44,45]. Couple discord has been shown to increase the likelihood of depressive symptoms, and at the same time, depressive symptoms have a deleterious effect on couple outcomes [43,46].

The association between depressive symptoms and couple discord can be a result of the burden experienced by the partner of the depressed person, and the reduction in income and in joint leisure activities stemming from the depression. Furthermore, the worry towards the depressed person, together with the withdrawal and the lack of support or interest of the depressed person are also proposed mechanisms that explain the harmful effects of depression on the couple’s relationship [43].

**Figure 1:**
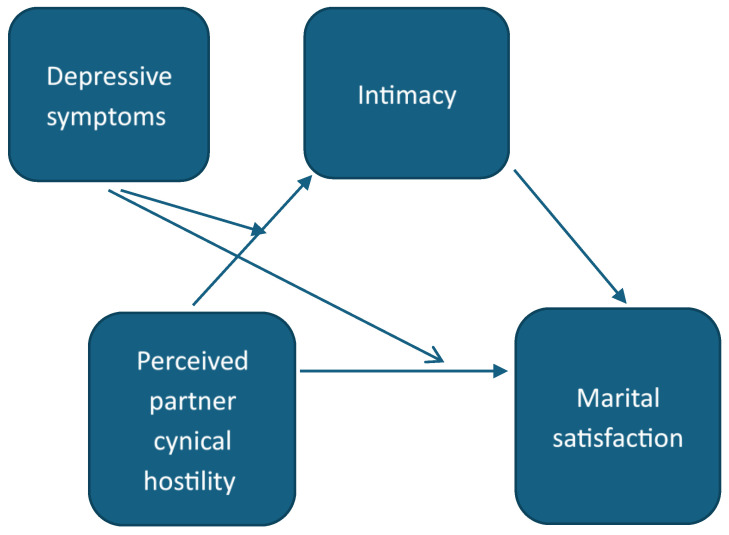
A theoretical model of the mediation role of intimacy in the relationship between perceived partner cynical hostility and marital satisfaction, and the moderating role of depressive symptoms.

**Figure 2:**
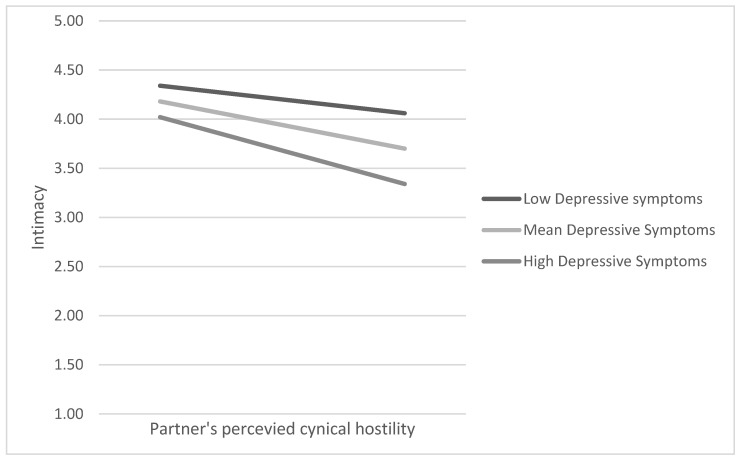
The moderating effect of depressive symptoms on the relationship between perceived partner cynical hostility and intimacy at T1.

Individuals whose partners are high in cynical hostility may be especially susceptible to the negative outcomes of their own depressive symptoms. A partner with high levels of cynical hostility may be less able or willing to provide support at times of need, such as during a lockdown [30,40], thus experiencing anxiety and withdrawing or responding aggressively when their partner experiences distress. These behaviors may not only exacerbate their partner’s experience of distress, but also have negative implications on the relationship, and first and foremost, the couple’s ability to experience intimacy. Thus, we hypothesize that depressive symptoms will moderate the relationship between a partner’s perceived cynical hostility and intimacy (H2), such that high levels of depressive symptoms will strengthen the negative association between cynical hostility and intimacy (Figure 1), and that depressive symptoms will moderate the relationship between a partner’s perceived cynical hostility and relationship satisfaction (H3), such that high levels of depressive symptoms will strengthen the negative association between cynical hostility and relationship satisfaction (Figure 2).

In applying the Vulnerability–Stress–Adaptation (VSA) model to our study, we emphasize the role of cynical hostility as a key enduring vulnerability that undermines couples’ adaptive processes, particularly intimacy. Cynical hostility fosters mistrust and negative expectations about others’ intentions, which can hinder emotional closeness and self-disclosure between partners. These effects are exacerbated under stressful conditions, such as the COVID-19 pandemic, when adaptive processes are critical to relationship stability. Depressive symptoms further compound this dynamic by intensifying the negative impact of perceived partner cynical hostility on intimacy. When one partner experiences emotional distress, the other’s cynical hostility may reduce their capacity to provide support or engage in intimate exchanges, deepening relationship dissatisfaction. Thus, cynical hostility and depressive symptoms interact to create a cycle of vulnerability that disrupts both intimacy and relationship satisfaction, aligning with the key principles of the VSA model.

The collection of two waves of data, one during a very stressful situation and one after it, could allow to us to examine whether the effects found are similar, or rather diverge between the waves, and to examine for the presence any long-term effects.

## 2. Materials and Methods

### 2.1. Participants

Data were collected from 309 eligible respondents during the first lockdown in Israel (March–April 2020) (T1). Participants included 60 men and 209 women (mean age = 44.42, SD = 13.48), who were recruited online via the authors’ social networks. Inclusion criteria were being over 18 and cohabiting with a romantic partner, either married (n = 248, 67%) or not. A proportion of 96% of the participants defined themselves as Jewish, with a mean of 17 years of education (SD = 3). The average length of the relationship was 18.9 years (SD = 14.87), and 64.8% had children living with them in the household. After providing informed consent, participants were directed into the main questionnaire. Our analytical sample includes 263 participants that provided complete data. Participants with complete data did not significantly differ from those who did not complete the questionnaire on any of the study’s variables. The second wave of data (T2) was collected soon as most of the restrictions were alleviated, in early June 2020. Participants were contacted and asked to fill out a second questionnaire initially intended to examine changes in their relationships. A total of 104 participants completed the second wave of data collection. Those who did not complete the second questionnaire had significantly fewer years of education (*M* = 16.59 vs. M = 17.07, t(274) = −3.51, *p* < 0.001). No other significant differences were found.

The study was approved by the Institutional Review Board. Due to a very strict timeline (the lockdown started without a warning and its expected duration was not known), the research was not pre-registered. All questionnaires were administered in Hebrew following a back-translation process conducted by two bilingual researchers.

### 2.2. Measurements

Relationship satisfaction. We measured relationship satisfaction using the Perceived Relationship Quality Components (PRQC) Inventory [47], which consists of seven items, rated on a scale ranging from 1 (“not at all”) to 5 (“very much”) (example item: “How much do you love your partner?”) (α = 0.94 at T1; α = 0.95 at T2).

Intimacy. Intimacy was assessed by using the emotional intimacy subscale from the Personal Assessment of Intimacy in Relationships (PAIR) inventory [48]. The subscale includes six items rated on a scale from 1 (“not at all”) to 5 (“very much”) (for example, “My partner can really understand my hurts and joys”) (α = 0.85 at T1; α = 0.85 at T2).

Cynical hostility. Perceived self and partner cynical hostility were measured using five items derived from the Cook–Medley Hostility Inventory [49,50], and used in the Health and Retirement Study. Respondents reported their level of agreement with the five items twice: once regarding themselves, and once regarding their partner (for example, “I commonly wonder what hidden reasons another person may have for doing something nice for me”, and “my partner commonly wonders what hidden reasons another person may have for doing something nice for him/her”). Items were rated on a scale ranging from 1 (“strongly disagree”) to 6 (“strongly agree”) (α = 0.77 for self; α = 0.84 for partner at T1; α = 0.84 for self; α = 0.89 for partner at T2).

Depressive symptoms. Depressive symptoms were measured using the relevant subscale from the Brief Symptom Inventory-18 (BSI-18) [51]. The depression subscale consists of nine items (for example, “Feeling no interest in things”), and participants are asked to report the frequency of occurrence during the last week on a scale ranging from 1 (“not at all”) to 5 (“all the time”) (α = 0.80 at T1; α = 0.84 at T2).

In addition, we controlled for respondent’s age, measured in years, and gender coded as 1 for men and 0 for women.

### 2.3. Analysis

To test the mediation hypothesis, we used regression in steps, in line with Baron and Kenny’s guidelines [52]. Accordingly, first, the dependent variable (relationship satisfaction) is regressed on the independent variable (cynical hostility) (Step 1); second, the mediator (intimacy) is regressed on the independent variable; third, the dependent variable is regressed on both the independent variable and the mediator to confirm that it is associated with the mediator and that the association between the independent variable and the dependent variable is reduced, thus suggesting mediation (Step 2). We used the Process package [53] to confirm the mediation using bootstrap analysis. To test the moderated mediation hypotheses, we included the interaction term between perceived partner cynical hostility and one’s own depressive symptoms in the equations assessing the relationship between perceived partner cynical hostility and intimacy, and between perceived partner cynical hostility and relationship satisfaction (see Figure 1). Similarly, we used the Process package to perform bootstrapping to estimate the moderated indirect effect [53]. The models were run separately for each wave, and longitudinal effects were examined in another model, where the T2 dependent variable was regressed on the T1 independent variable while controlling for the T1 level of the dependent variable.

## 3. Results

Descriptive statistics and correlations between all the study variables are presented in Table 1. Whereas one’s own cynical hostility is not significantly correlated with neither intimacy nor relationship satisfaction at T1, the perceived partner cynical hostility was negatively correlated with both (r = −0.27, *p* < 0.001 for intimacy; r = −0.27, *p* < 0.001 for relationship satisfaction). One’s own cynical hostility was significantly correlated with intimacy at T2 (r = −0.31, *p* < 0.001) and with relationship satisfaction (r = −0.25, *p* < 0.001). The relationship satisfaction remained very stable between the two waves (r = 0.85, *p* < 0.001), implying that finding longitudinal effects is highly unlikely.

Due to the high correlation between intimacy and relationship satisfaction, we performed a confirmatory factor analysis, testing two models: one with all the items loaded into one factor, and a second with the items loaded into their deferential variables. The two-factor model showed a significantly better fit to the data, supporting the two-factor solution (χ^2^(65) = 316.64 for the one-factor model; χ^2^(64) = 196.16 for the two-factor model).

To test the first hypothesis, according to which intimacy will mediate the association between one’s own (H1a) and partner’s (H1b) cynical hostility and relationship satisfaction, we used the Process package 3.5. The initial regression analysis (Table 2) showed that one’s own cynical hostility was not significantly associated with the outcome variable, neither at T1 nor at T2, and, hence, in the mediation analysis, we focused on the perceived partner cynical hostility. The perceived partner cynical hostility and the participant’s own depressive symptoms were centered around the mean before their inclusion in the analysis. At T1, the perceived partner cynical hostility was a significant correlate of intimacy (*b* = −0.29, se = 0.07, *p* < 0.001; not in Table 2), and of relationship satisfaction (*b* = −0.22, se = 0.05, *p* < 0.001; Step 1 Table 2). When adding intimacy to the equation predicting relationship satisfaction (Step 2), the association between the perceived partner cynical hostility becomes insignificant: the direct effect between the perceived partner cynical hostility and relationship satisfaction when accounting for intimacy is b = −0.04, se = 0.03, *p* = ns. Using 5000 bootstrap samples and with 95% confidence intervals, we found that the indirect effect was of *b* = −0.17, se = 0.04, LLCI = −0.25, ULCI = −0.10. This suggests full mediation, where the mediation effects account for 81% of the total effect. Thus, H1b was supported. The results were replicated using the T2 measurements, such that the perceived partner cynical hostility was significantly associated with intimacy (*b* = −0.27, se = 0.12, *p* < 0.05) and with relationship satisfaction (b = −0.25, se = 0.12, *p* < 0.05). Similarly to T1, intimacy significantly mediated the association between the perceived partner cynical hostility and relationship satisfaction (*b* = −0.22, se = 0.11, LLCI = −0.45, ULCI = −0.03). Longitudinally, the mediating effect of intimacy remained significant when regressing T2 relationship satisfaction on T1 independent and mediator variables, but became insignificant when T1 relationship satisfaction was added to the equation as a control variable to test for a residual change in relationship satisfaction. That is, the T1 variables were not predictive of T2 relationship satisfaction when taking into account the initial levels of satisfaction.

**Figure 3:**
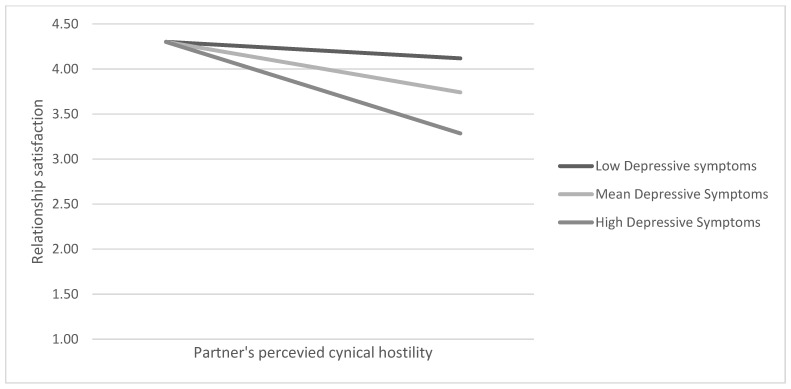
The moderating role of depressive symptoms in the relationship between perceived partner cynical hostility and relationship satisfaction at T2.

In the next step, we estimated whether one’s depressive symptoms moderated the relationship between the perceived partner cynical hostility and intimacy (H2), and between the perceived partner cynical hostility and relationship satisfaction (H3) (see Figure 1). In support of H2, we found a significant moderation of the association between the perceived partner cynical hostility and intimacy at T1 (*b* = −0.04, se = 0.02, *p* < 0.05). A simple slopes analysis showed that the association between the perceived partner cynical hostility and intimacy was stronger when depressive symptoms were more prevalent (*b* = −0.17, se = 0.09, *p* < 0.10 when depressive symptoms were 1 SD below the mean; *b* = −0.29, se = 0.06, *p* < 0.001 when depressive symptoms are at the mean; *b* = −0.41, se = 0.09, *p* < 0.001 when depressive symptoms are 1 SD above the mean) (Figure 2). Depressive symptoms did not, however, moderate the association between the perceived partner cynical hostility and relationship satisfaction, and were omitted from the equation. Thus, the indirect effect estimates for the moderated mediation model were as follows: *b* = −0.10, se = 0.05, LLCI = −0.21, ULCI = −0.01 for low levels of depressive symptoms; *b* = −0.17, se = 0.04, LLCI = −0.25, ULCI = −0.10 for mean levels of depressive symptoms; and *b* = −0.24, se = 0.06, LLCI = −0.36, UPLCI = −0.13. H2 was supported and H3 was not. In T2, depressive symptoms significantly moderated the associations between the perceived partner cynical hostility and intimacy (b = −0.07, se = 0.03, *p* < 0.05; conditional effects: b = −0.10, se = 0.13, *p* = ns when depressive symptoms are low; b = −0.31, se = 0.12, *p* < 0.01 when depressive symptoms are at the mean; b = −0.56, se = 0.17, *p* < 0.01 when depressive symptoms are high), and the associations between the partner’s hostility and relationship satisfaction (b = −0.05, b = 0.02, *p* < 0.05; conditional effects: b = 0.04, se = 0.08, *p* = ns when low; b = −0.09, se = 0.07, *p* = ns when depressive symptoms at mean; b = −0.25, se = 0.11, *p* < 0.05 when depressive symptoms are high). The conditional indirect effect was as follows: b = −0.07, LLCI = −0.23, ULCI = 0.09 when depressive symptoms are low; b = −0.23, LLCI = −0.40. ULCI = −0.06 when depressive symptoms are at the mean; b = −0.41, LLCI = −77, ULCI = −0.12 when depressive symptoms are high) (Figure 3). No longitudinal effects were found.

We further tested whether the age or relationship length moderated the model, and found no effect. Testing the model separately for men and women, the results were significant for women, but not for men. However, given the limited number of men in the sample, we believe that the insignificance of the findings reflects the lack of statistical power. Future research would need to gather a larger sample.

## 4. Discussion

Within the context of COVID-19, the goals of the current study were twofold. First, we nominated cynical hostility—the perception that people are a source of wrongdoing and cannot be trusted—as one enduring vulnerability factor that explains decreased relationship intimacy and satisfaction during the pandemic. Our second objective was to explore the role of depressive symptoms in moderating the association between the perceived partner cynical hostility and couples’ intimacy and relationship satisfaction. Our results pointed to the significant role played by one’s perceived partner cynical hostility in predicting couples’ intimacy and relationship satisfaction. These findings add to those of previous studies emphasizing the toll that perceived partner cynical hostility takes on individuals’ mental health and social relationships [26,27,28].

The Vulnerability–Stress–Adaptation (VSA) model suggests that couples’ responses to stress are influenced by enduring vulnerabilities, such as cynical hostility, which affect relationship processes like intimacy and satisfaction. The association between the perceived partner cynical hostility and relationship satisfaction was mediated by the couple’s intimacy, suggesting that cynical hostility particularly harms the ability of romantic partners to co-construct closeness in times of need, which is an adaptive process, according to the VSA, with meaningful implications for relationship satisfaction. Our results also highlighted the significance of depressive symptoms in this process, which can be conceptualized as an additional vulnerability factor. Depressive symptoms were found to moderate the association between the perceived partner cynical hostility and couple intimacy, and, though less conclusively, between cynical hostility and relationship satisfaction.

In a time of a global pandemic with ongoing uncertainty and intense change, intimacy can constitute an adaptive process for couples that helps them to adjust to the stressful reality. Couple intimacy, thus, could have been a source of resilience for partners during the COVID-19 global pandemic whereas low levels of intimacy can reflect as well as generate distress within the partnership, resulting in decreased relationship satisfaction, that can, in turn, threaten the stability of the relationship. More specifically, when one perceives his or her partner as holding cynically hostile beliefs, that is, expressing a fundamental lack of trust in others’ intentions, they may become less willing to invest in creating closeness. Feeling they cannot self-disclose, individuals may avoid sharing their fears and concerns with their partners, leaving them experiencing relational distance. Furthermore, a partner with high levels of cynical hostility may lack the ability to empathically and supportively [54] respond to the closeness-seeking attempts of the spouse, responding with disrespect [19], impatience, or aggression, thus also contributing to a perceived lack of intimacy. This lack of ability to form a secure and intimate relationship may be especially harmful when one needs it most, that is, when they experience emotional distress that can especially prominent in difficult times, such as the pandemic. 

The experience of emotional distress can be perceived as especially threatening to partners that are high in cynical hostility, because the need to provide support may be stressful for them [30]. In reaction to their partner’s distress, individuals with higher levels of cynical hostility may respond with more hostile behavior, and as a result, the perceived gap between their partner’s support needs and the support gained within the relationship is expanded.

Data for the current study were collected during the peak of the first wave of COVID-19 in Israel, when a strict lockdown was enforced, and a sense of danger and anxiety were largely prevalent [55], and immediately after most of the restrictions were alleviated. Whereas individuals with lower levels of cynical hostility may be more able to seek support within their social networks, those with higher levels of cynical hostility may not only find themselves in the uncomfortable position of support provision, but also lacking a supportive network of their own [25,29,30]. These two concurrent processes could result in more hostile interpersonal behavior [29], creating more conflicts within the dyad [54], and preventing the couple from achieving intimate and supportive interactions.

Overall, whereas most previous studies have focused on the toll that cynical hostility takes on the individual [25,30], the current study joins the growing body of research that looks at the toll that perceived cynical hostility takes on relational partners [19,26,29]. Specifically, we suggest that it is those who live with partners who are perceived to have higher levels of cynical hostility that may be particularly vulnerable. Their vulnerability may become even more apparent when they experience personal hardships, measured here in the form of depressive symptoms. At the dyadic level, their partner’s inability to adequately respond to their emotional needs may contribute to the increased distance between them and to decreased positive marital interactions [56], possibly leading to relationship dissatisfaction and instability [1].

Surprisingly, our results only partly supported our hypothesis that depressive symptoms would moderate the association between perceived partner cynical hostility and relationship satisfaction. That is, the moderating effect was found only in the second wave of data collection (after the lockdown). When individuals experience distress, the ability to closely and intimately interact with someone is crucial. When their partners are high in cynical hostility, this ability is severely hindered. However, other positive relational aspects, such as the enjoyment of time together and practical support, may not be as susceptible to the additive effects of perceived partner cynical hostility and one’s own depressive symptoms, at least when the external circumstances are highly threatening, and require close proximity. However, as things went back to normal, we found that, indeed, those whose depressive symptoms lingered and their symptoms were high suffered from lower satisfaction when their partner was perceived as high in cynical hostility.

We also did not find that one’s own cynical hostility was related to couple intimacy. A possible explanation is that individuals with higher levels of cynical hostility may be less attuned to couple adaptive processes, such as intimacy. Possibly, such individuals are not as attentive to their partner’s needs and expressions of dissatisfaction as individuals with low cynical hostility, and, hence, the lack of association between one’s own cynical hostility and intimacy. Although this finding is in somewhat contradiction to previous findings that documented associations between one’s cynical hostility and relationship quality [35], it is in line with MacKenzie et al. [36], who did not find an association between cynical hostility and emotional expression. It may be that it is the responsiveness component that is missing when trying to create intimacy [22], and this should be further examined in future research.

### Limitations, Future Directions, and Implications of the Study

The current study has several limitations that should be noted. First, the convenience sampling method limits the ability to generalize the findings. As the researchers used mainly their own social networks, the sample, which is mostly Jewish and highly educated, does not represent the Israeli population. Future research should recruit a more representative sample. Another limitation is that the data were not directly collected from dyads. Rather, the individuals rated their perceived partner cynical hostility, which may represent the rater’s bias and not necessarily the partner’s cynical hostility. Future research should test this model longitudinally using dyadic data. We also did not find any longitudinal findings, implying that causality cannot be determined. Although we suggested that cynical hostility is the driving force behind lower levels of intimacy and relationship satisfaction, it is also possible that a lack of intimacy increases individuals’ views of their partners as high in cynical hostility. Previous studies suggest that both directions of causality are possible [19,25], and the reciprocal effects of intimacy and cynical hostility in couples should be examined in future research. The lack of longitudinal findings could also be due to the small sample obtained, which is not sufficient to detect small effects, and the model should be tested using larger longitudinal samples. Furthermore, future research could examine other possible outcomes, such as couple conflict and relationship stability, as well as other forms of intimacy, such as sexual and social intimacy. Third, although the study was specifically designed to assess couples’ relationship during the first COVID-19 lockdown, the measurements were not specific to COVID-19. However, we believe that the results reflect individuals’ responses to the extreme situation, in line with other studies, identifying high levels of anxiety and depression following the COVID-19 outbreak [4]. Finally, there are limitations on generality due to the cultural context and the specific historical time in which the data were collected.

Despite these limitations, this study has several important theoretical and practical contributions. Theoretically, the study highlights the role of perceived partner cynical hostility as an enduring vulnerability factor that shapes couples’ adaptive processes (intimacy) and couple outcomes (satisfaction) [1]. As depressive symptoms may not only cause significant distress, but also reduce individuals’ ability to mobilize support from their social networks, depressed individuals living with partners with high levels of cynical hostility may be even more vulnerable, especially in times of stress such as during the COVID-19 pandemic, as their ability to gain intimacy within the romantic relationship was significantly decreased. During this time of the global pandemic, where families faced meaningful health threats, coupled with changed routines that often forced them to spend more time together and reduce their contact with others who did not share their household, understanding how partners’ characteristics shape adaptive processes is of importance. Practitioners providing support to couples coping with difficulties should take into account partners’ cognitive schemas, such as cynical hostility. Even if this cognitive schema is not directed at the intimate partner, the cynical beliefs may still shape dyadic processes within the relationship. The toll that these partners’ beliefs take on the relationship may be accentuated in times of external stress and personal difficulties.

## Figures and Tables

**Table 1:** Descriptive statistics and correlations between study variables (N = 263).

	M	SD	1	2	3	4	5	6	7	8	9	10	11
1. Age	44.50	13.48											
2. Gender	0.20	0.40	0.05										
3. T1 hostility	20.74	0.91	−0.11	0.19 **									
4. T1 partner hostility	20.40	0.83	0.04	0.04	0.45 ***								
5. T1 depressive symptoms	90.60	30.40	−0.25 ***	−0.02	0.16 **	0.04							
6. T1 intimacy	30.94	0.81	−0.03	0.001	−0.09	−0.27 ***	−0.28 ***						
7. T1 relationship satisfaction	40.41	0.64	0.05	0.001	−0.10	−0.27 ***	−0.27 ***	−0.76 ***					
8. T2 hostility	20.80	10.07	−0.06	−0.12	0.73 ***	0.38 ***	0.20 *	−0.06	−0.11				
9. T2 partner’s hostility	20.55	0.91	−0.05	0.01	0.46 ***	0.58 ***	0.12	−0.22 *	−0.21 *	0.67 ***			
10. T2 depressive symptoms	90.09	30.70	−0.07	−0.18	0.12	0.09	0.54 ***	−0.21 *	−0.30 **	0.27 **	0.21 *		
11. T2 intimacy	30.98	0.83	−0.01	0.05	−0.23 *	−0.30 **	−0.31 **	0.77 ***	0.75 ***	−0.23 *	−0.31 ***	−0.36 ***	
12. T2 relationship satisfaction	40.37	0.81	0.09	−0.01	−0.13	−0.22 *	−0.31 ***	0.70 ***	0.85 ***	−0.14	−0.25 *	−0.38 ***	0.81 ***

* *p* < 0.05, ** *p* < 0.01, *** *p* < 0.001.

**Table 2:** Regression analysis predicting relationship satisfaction from perceived partner cynical hostility and intimacy.

	T1 (N = 263)				T2 (N = 104)			
	Step 1		Step 2		Step 1		Step 2	
Variable	b	se	b	se	b	se	b	se
Intercept	4.19 ***	0.20	1.86 ***	0.19	4.68	0.37	0.81	0.36
Age	0.00	0.00	0.00	0.00	0.01	0.01	0.01	0.00
Gender	0.00	0.10	−0.01	0.07	0.01	0.19	0.09	0.11
Own hostility	0.03	0.05	0.01	0.03	0.05	0.10	0.07	0.07
Partner’s hostility	−0.22 ***	0.05	−0.04	0.04	−0.25 *	0.12	-0.05	0.07
Intimacy			0.60 ***	0.03			0.80 ***	0.06
R^2^	0.07		0.60		0.07		0.68	
ΔR^2^			0.53 ***				0.61 ***	

* *p* <0.05, *** *p* < 0.001.

## Data Availability

The original contributions presented in the study are included in the article, further inquiries can be directed to the corresponding author.
